# Upregulated Expression of ErbB1 in Diffuse Large B-Cell Lymphoma as a Predictor of Poor Overall Survival Outcome

**DOI:** 10.3390/jpm13050770

**Published:** 2023-04-29

**Authors:** Sanjive Qazi, Fatih M. Uckun

**Affiliations:** Immuno-Oncology Program, Ares Pharmaceuticals, St. Paul, MN 55110, USA

**Keywords:** ERBB1, EGF, protein tyrosine kinase, diffuse large B-cell lymphoma, transcription factor

## Abstract

We examined the transcript-level expression of ErbB family protein tyrosine kinases, including ERBB1, in primary malignant lymphoma cells from 498 adult patients with diffuse large B-cell lymphoma (DLBCL). ERBB1 expression in DLBCL cells was significantly higher than in normal B-lineage lymphoid cells. An upregulated expression of ERBB1 mRNA in DLBCL cells was correlated with an amplified expression of mRNAs for transcription factors that recognized ERBB1 gene promoter sites. Notably, amplified ERBB1 expression in DLBCL and its subtypes were associated with significantly worse overall survival (OS). Our results encourage the further evaluation of the prognostic significance of high-level ERBB1 mRNA expression and the clinical potential of ERBB1-targeting therapeutics as personalized medicines in high-risk DLBCL.

## 1. Introduction

ERBB1 (also known as EGFR) is broadly expressed in numerous cell types of epithelial or mesenchymal origin [1,2,3]. ERBB1 signaling plays a vital role in regulating cell proliferation, differentiation, survival, and motility, as well as de-differentiation and malignant transformation [1,2,3,4,5,6]. It serves as a receptor for several ligands, such as the epidermal growth factor (EGF) and amphiregulin (AREG) [1,2,3,4,5,6]. The engagement of ERBB1 by one of its ligands results in the activation of its catalytic protein tyrosine kinase (PTK) function and triggers the tyrosine phosphorylation of the related receptor family of PTK, including ERBB2, ERBB3, and ERBB4 [1,2,3]. ERBB1 is overexpressed and/or intrinsically overactive due to mutations in malignant solid tumors [7,8,9,10,11]. Studies examining ERBB1 overexpression and/or overactivation demonstrated significant relationships in disease progression, fast invasive growth, and metastatic spread [7,8,9,10,11], as well as resistance to biotherapeutic drugs targeting inhibitor immune checkpoints [12].

While the expression and function of ERBB1 have been extensively studied in normal and malignant cells of epithelial and mesenchymal origins, very little is understood regarding its expression in normal or malignant lymphoid cells. Intriguingly, Zaiss et al. reported that CD4^+^ T-cells express ERBB1 in an antigen-dependent manner [13], which enables them to mount an innate-like immune response during gastrointestinal helminth infections [14]. ERBB1 signaling pathways play key roles in regulating T-cell homeostasis [15], and they may also influence the pathophysiology of atherosclerosis as a T-cell-mediated inflammatory disease [16]. ERBB1 expression was detected in a B-ALL cell line and shown to promote stroma-independent survival and growth [17]. AREG [18] as well as EGF [19] are capable of supporting the proliferation and survival of multiple myeloma (MM) cells upon binding to their ERBB1. Additionally, ERBB1 pathway activation by AREG in MM-derived exosomes has been reported to trigger osteoclastogenesis, and ERBB1 pathway inhibitors are cytotoxic to MM cells [20,21]. Our recent studies demonstrated that ERBB1 expression was upregulated in MM cells, and higher levels of ERBB1 were associated with poor treatment outcomes and survival in newly diagnosed MM [22].

In the present study, we examined the transcript-level expression of ERBB1 in neoplastic cells from patients with the most common type of adult B-lineage lymphoid malignancies, namely diffuse large B-cell lymphoma (DLBCL). Notably, ERBB1 messenger ribonucleic acid (mRNA) was overexpressed in malignant lymphoma cells (MLC) from DLBCL patients, especially those with the high-risk activated B-cell (ABC)-subtype. Upregulated expression of ERBB1 in DLBCL cells was correlated with an amplified expression of transcription factors (TF) that recognized ERBB1 gene promoter sites. Notably, amplified ERBB1 expression in DLBCL and its subtypes was associated with significantly worse overall survival (OS). 

## 2. Materials and Methods

### 2.1. Normalization of Gene Expression Data for DLBCL Samples

Three archived datasets deposited as CEL files were downloaded from the GEO repository (GSE10846, GSE12195, and GSE12453; https://www.ncbi.nlm.nih.gov/geo/; accessed on 30 July 2022) and processed to create an in-house probeset-level mRNA expression working database of primary MLC isolated from lymph node specimens of newly diagnosed patients with DLBCL (N = 498, GSE10846, GSE12195, and GSE12453). In addition, we used the archived dataset from GSE12195 and GSE12453 for normal B-lineage lymphoid cell (BLC) populations, including CD77^+^ germinal center centroblasts isolated by magnetic cell separation from normal human tonsils (N = 5), CD10^+^CD77^−^ germinal center centrocytes isolated by magnetic cell separation from normal human tonsils (N = 5), sIgD^+^CD27^−^CD10^−^CD38^−^ naïve B-cells isolated by magnetic cell separation from normal tonsils (N = 5), CD27^+^CD10^−^CD38^−^ memory B-cells isolated by magnetic cell separation from normal tonsils, FACS-sorted CD20^low^CD38^high^ plasma cells isolated from normal tonsils (N = 5), FACS-sorted sIgD^+^CD27^−^ naïve B-cells isolated from the peripheral blood of healthy donors (N = 5), FACS-sorted CD20^+^CD27^+^ memory B cells isolated from the peripheral blood of healthy donors (N = 5), FACS-sorted CD20^+^CD38^+^CD77^−^ centrocytes from normal tonsils (N = 5), and FACS-sorted CD20^+^CD38^+^CD77^+^ centroblasts isolated from normal tonsils (N = 5). Subsets of DLBCL patients were also used in our comparisons for gene expression and ERBB1-associated OS outcomes from the GSE10846 dataset. There were 188 limited-stage (Stage I or Stage II) patients and 218 advanced-stage (Stage III or Stage IV) patients. A total of 167 patients had ABC-type DLBCL, 183 had germinal center B-cell (GCB)-type DLBCL, and 64 had unclassified DLBCL. A normalization procedure performed at the level of each probeset enabled us to compare the mRNA expression of specific probesets in primary DLBCL cells vs. normal BLC populations from healthy volunteer donors. Batch normalized Affymetrix CEL files employing Aroma Affymetrix statistical packages ran in R quantified log_2_-scaled probeset-level PM signals using the method of Robust Multiarray Analysis as previously described [22]. 

We also examined ERBB1 expression levels (ERBB1 probesets 1565484_x_at and 1565483_at) in MLC from DLBCL patients with early treatment failures and fatal outcomes within 2.5 years. An analysis of variance (ANOVA) model with mixed effects was employed to compare the differential expression of ERBB1 mRNA in MLC from DLBCL patients who died within 2.5 years vs. DLBCL patients who remained alive at 2.5 years. Least square means and variance calculations from the “probeset × patient outcome group” interaction group were used for pairwise comparisons of patients experiencing death vs. alive patients at a follow-up time of 2.5 yrs. The genechip variance component was also considered in the model’s random factor. Cluster figures were constructed to display mRNA expression levels in DLBCL patients experiencing death divided by the mean expression levels of patients who remained alive at 2.5 years follow-up. 

### 2.2. Hierarchical Clustering Analysis

A hierarchical clustering technique organized probeset-level and patient-level mRNA profiles to visualize similarly expressing samples and probesets by clustering them together using the average distance metric to the mRNA expression profiles as previously described in detail [22].

### 2.3. Statistical Methods for the Analysis of Differential mRNA Expression

Differential mRNA expression was analyzed, as previously described in detail [22]. ANOVA models with mixed effects were carried out in the R, as previously described [22], to determine the log_2_-transformed fold changes for probesets in group comparisons. *p*-values less than 0.05 were deemed significant (FDR < 0.1) using linear contrasts between the comparison groups, as previously described [22].

ERBB1 mRNA levels in DLBCL cells were correlated with mRNA levels of 20 TF, which recognized ERBB1 gene promoter sites across 414 adult DLBCL patients (a total of 60 probesets on the Affymetrix gene chip), as previously reported for MM cells [22]. These TF were: Beta-catenin (CTNNB1), Fos Proto-Oncogene, AP-1 TF subunit (FOS/AP-1), FosB Proto-Oncogene, AP-1 TF subunit (FOSB/AP-1), FOS Like 1, AP-1 TF subunit (FOSL1/FRA-1/AP-1), FOS Like 2, AP-1 TF subunit (FOSL2/FRA-2/AP-1), GC-Rich Sequence DNA-Binding factor 2 (GCFC2/TCF9), Homeobox B5 (HOXB5), Interferon regulatory factor 1 (IRF1), Jun Proto-Oncogene, AP-1 TF subunit (JUN/AP-1), Sp1 TF (SP1), Tumor Protein P53 (TP53), TEA domain TF 2 (TEAD2/ETF), TATA-Box Binding Protein (TBP), POU Class 6 Homeobox 2 (POU6F2/RPF-1), JunD Proto-Oncogene, AP-1 TF subunit (JUND/AP-1), Lymphoid Enhancer Binding Factor 1 (LEF1), TF 7 (TCF7), TF7 Like 1 (TCF7L1), TF 7 Like 2 (TCF7L2), and JunB Proto-Oncogene, AP-1 TF subunit (JUNB/AP-1) [23,24,25,26,27,28,29,30,31,32,33,34,35,36,37,38,39,40]. Pairwise correlation coefficients across all 60 probesets were presented as a square matrix (3600 comparisons (60 × 60), of which 60 were self-comparisons; 3540 correlation coefficients were used for false discovery rate calculations). To identify the clusters of co-regulated probesets, a clustering technique was used to group the probesets and visualize the relations using heatmaps (red to blue representing correlation coefficients from +1 to -1, respectively) (as previously described [22]). The null hypothesis to reject or accept a zero-correlation coefficient was adapted from T-tests (*p* < 0.05 and false discovery rate (FDR) < 0.1). A total of 2738 significant correlations were identified (*p* < 0.05 and FDR = 0.05), and 2092 comparisons resulted in highly significant correlations (*p*-values < 0.0001 and FDR < 0.001).

### 2.4. Survival Analysis in Relationship to ERBB1 Expression

The impact of the transcript level overexpression of ERBB1 in primary MLC on OS was examined using the archived treatment outcome and survival data from GSE10846 for 414 DLBCL patients treated with chemotherapy +/− Rituximab, including 181 CHOP-treated patients and 233 Rituximab + CHOP-treated patients [41,42]. The same type of analysis was also performed for a subset of 231 non-GCB-type (ABC-type or unclassified) DLBCL patients (Pooled 167 ABC-type DLBCL patients with 64 Unclassified patients) as well as 218 advanced-stage DLBCL patients (Pooled 97 Stage 3 patients and 121 Stage 4 patients). In our investigation of the OS outcomes, we used the Kaplan–Meier (KM) method to calculate survival probabilities and to detect the differences in survival times between the comparison groups (Log-rank chi-squared tests, *p* < 0.05 deemed significant) as previously described in detail [22]. 

### 2.5. Processing and Analysis of the RNA-seq-Based Validation Dataset from the TCGA Project

Archived data on the RNA Sequencing (RNA-seq)-based mRNA expression profiles of MLC from 47 DLBCL patients were obtained as a validation dataset from the Cancer Genome Atlas (TCGA), accessed on 12 March 2023), and normalized using the University of California Santa Cruz XENA platform (https://xenabrowser.net/; accessed on 12 March 2023), as described [43]. We used the Gene Expression Profiling Interactive Analysis tool, GEPIA2 (http://gepia2.cancer-pku.cn, accessed 12 March 2023) via the web portal to examine the effects of RNAseq-based intra-tumor ERBB1 mRNA levels on the DFS and OS outcomes of DLBCL patients, as previously described for MM patients [22]. GEPIA2 provided the Kaplan–Meier survival analysis for the EGFR/ERBB1^high^ (viz.: 18 patients with ERBB1 mRNA expression levels greater than or equal to the top 40th percentile of ranked values) and EGFR/ERBB1^low^ patient group (viz.: 18 patients with the ERBB1 mRNA expression levels less than or equal to the bottom 40th percentile of ranked values) comparisons. Hazard ratios for the EGFR/ERBB1^high^ group were calculated for DFS/OS using a univariate Cox proportional hazards model for these 2 patient groupings. We tested whether the DFS and OS probabilities for the EGFR/ERBB1^low^ at 100 months were greater than the upper 95% confidence interval for the DFS and OS probabilities for the EGFR/ERBB1^high^ group of patients. 

## 3. Results

### 3.1. Amplified Transcript-Level Expression of ERBB1 in MLC from DLBCL Patients

**Figure 1:**
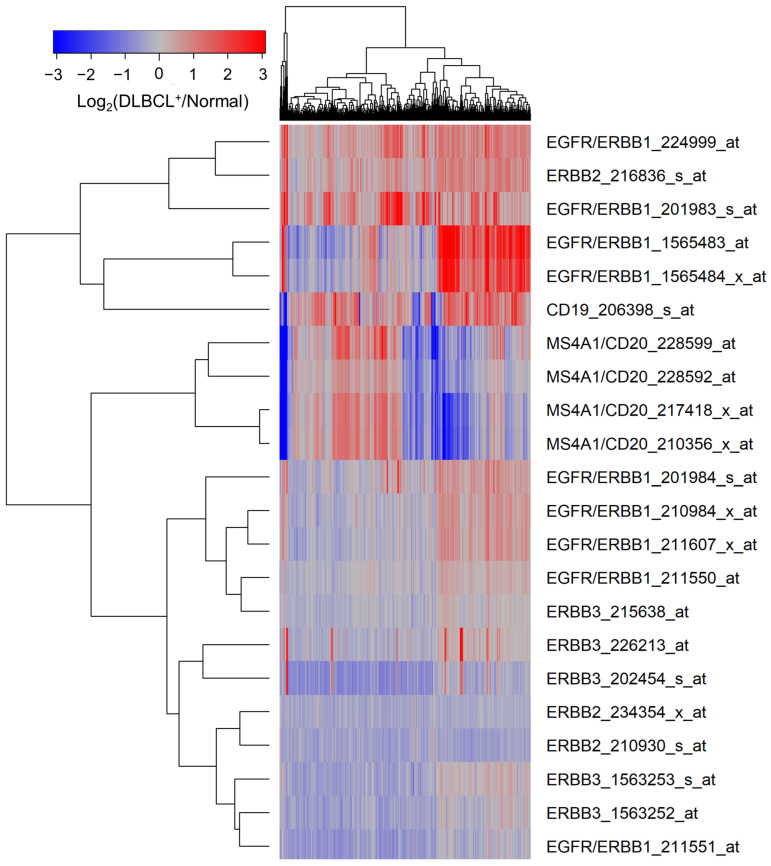
Upregulated expression of ERBB1 mRNA in MLC from adult DLBCL patients. Depicted is a cluster figure of the mRNA expression levels for ERBB1, ERBB2, and ERBB3 as well as B-lineage surface receptors CD19 and CD20 in MLC from DLBCL patients (N = 498) mean centered to the corresponding mRNA expression levels in normal BLC (N = 45). The cluster figure shows the log_2_-transformed fold-change values (blue represents underexpression, and the red color represents overexpression in samples from DLBCL patients). ERBB1 mRNA was significantly upregulated (Probeset ERBB1_201983_s_at: Fold Change = 1.94; *p*-value < 10^−8^; Probeset ERBB1_224999_at: Fold Change = 1.74; *p*-value < 10^−8^; Probeset ERBB1_1565483_at: Fold Change = 1.72; *p*-value < 10^−8^) (Supplementary Materials: Table S1).

MLC from 498 newly diagnosed DLBCL patients expressed significantly higher levels of ERBB1 mRNA than normal BLC populations in 45 healthy volunteers (Figure 1, Supplementary Materials: Table S1). mRNA levels measured using the probeset ERBB1_201983_s_at exhibited the greatest fold change (Fold Change = 1.94; *p*-value < 10^−8^) (Supplementary Materials: Table S1). CD19 mRNA was also expressed at a 1.54-fold higher level in the MLC from DLBCL patients compared to BLC (Figure 1, Supplementary Materials: Table S1).

Transcriptomes of MLC from GCB-type DLBCL patients (N = 183) were compared to those of MLC from non-GCB (ABC-type or unclassified) type DLBCL patients (N = 231), as well as the normal BLC in the control samples (N = 45). MLC in each DLBCL subset exhibited an amplified ERBB1 expression compared to normal control BLC populations, and the highest expression levels were observed in the non-GCB subset (Supplementary Materials: Figures S1–S4, Tables S2–S5). 

**Figure 2:**
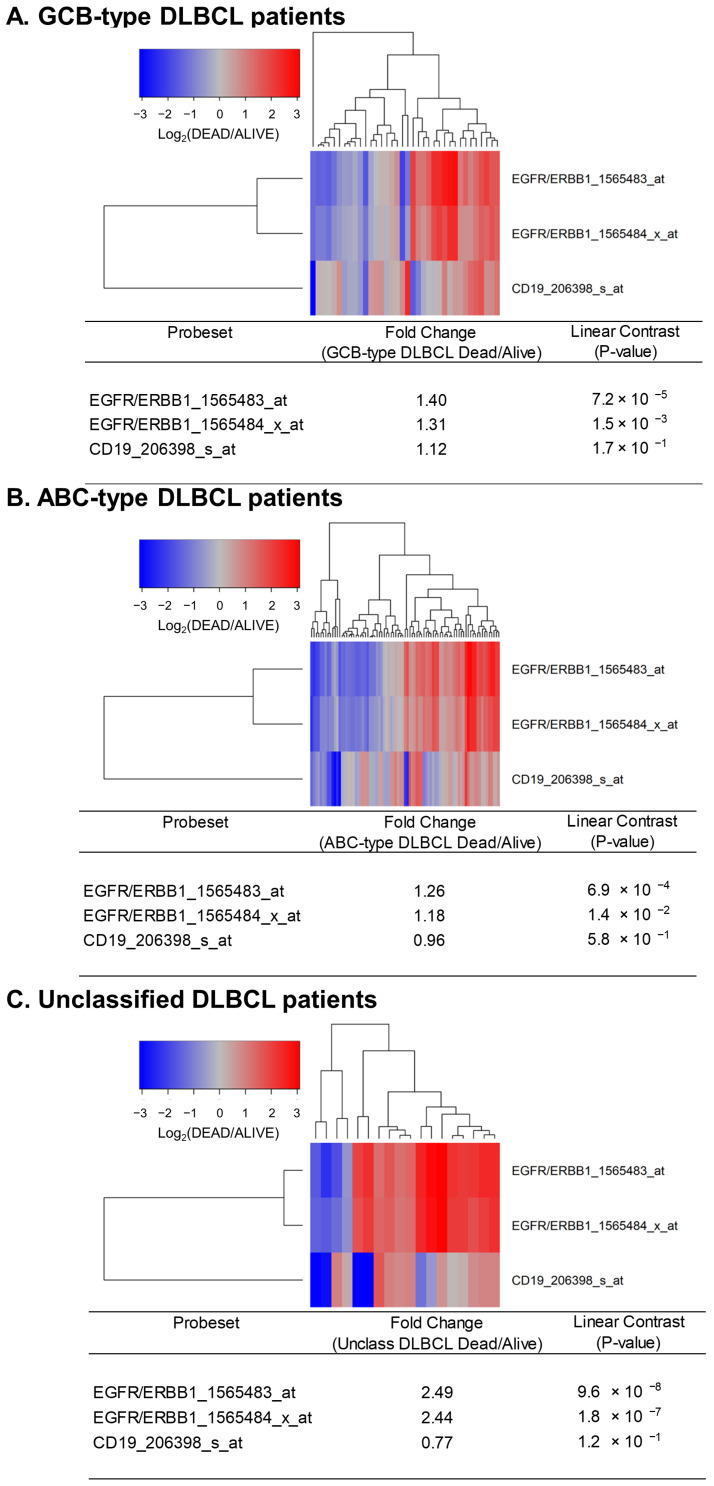
Upregulated expression of ERBB1 mRNA in MLC from DLBCL patients who experienced early treatment failures and fatal outcomes. Depicted are cluster figures of the mRNA expression levels for ERBB1 as well as B-lineage surface receptor CD19 in the MLC from DLBCL patients who died within 2.5 years when mean centered to the corresponding mRNA expression levels in the MLC from DLBCL patients who remained alive after 2.5 years. The cluster figure shows the log_2_-transformed fold-change values (blue represents underexpression, and the red color represents overexpression in MLC from patients experiencing early death). (**A**) At 2.5 yrs, 36 of 183 GCB-type DLBCL patients had died, and 147 remained alive. GCB-type DLBCL patients who died within 2.5 years exhibited an increased expression of ERBB1 mRNA (but not CD19 mRNA) compared to GCB-type DLBCL patients who remained alive after 2.5 years (Probeset EGFR/ERBB1_1565483_at: 1.40-fold increased expression, *p*-value = 7.2 × 10^−8^; Probeset EGFR/ERBB1_1565484_x_at: 1.31-fold increased expression, *p*-value = 1.5 × 10^−3^). (**B**) At 2.5 yrs, 81 of 167 ABC-type DLBCL patients had died, and 86 remained alive. ABC-type DLBCL patients who died within 2.5 years exhibited an increased expression of ERBB1 mRNA (but not CD19 mRNA) compared to ABC-type DLBCL patients who remained alive after 2.5 years (Probeset EGFR/ERBB1_1565483_at: 1.26-fold increased expression, *p*-value = 6.9 × 10^−4^; Probeset an EGFR/ERBB1_1565484_x_at: 1.18-fold increased expression, *p*-value = 1.4 × 10^−2^) (**C**) Compared to the MLC from 46 unclassified DLBCL patients who remained alive after 2.5 years, MLC from 18 unclassified DLBCL patients who died within 2.5 years showed an increased ERBB1 mRNA expression (but not CD19 mRNA) (Probeset EGFR/ERBB1_1565483_at: 2.49-fold increased expression, *p*-value = 9.6 × 10^−8^; Probeset EGFR/ERBB1_1565484_x_at: 2.44-fold increased expression, *p*-value = 1.8 × 10^−7^).

**Figure 3:**
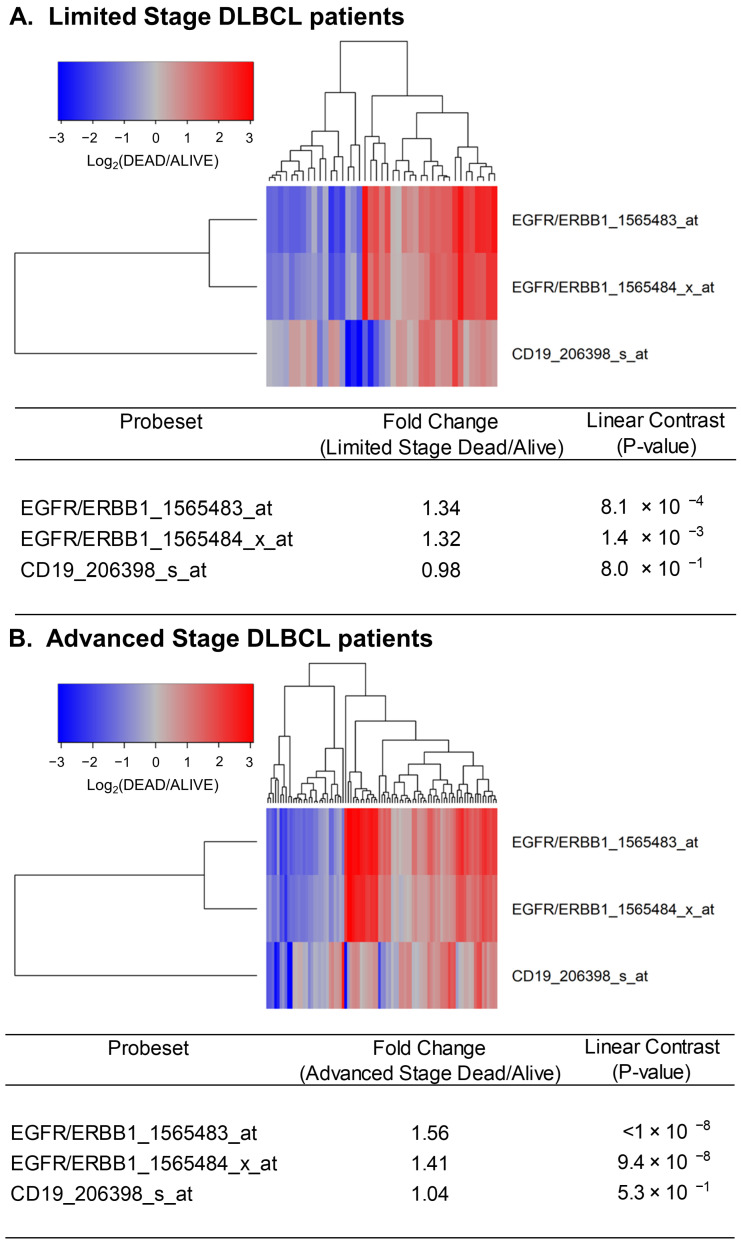
Upregulated expression of ERBB1 mRNA in MLC from limited-stage and advanced-stage DLBCL patients who experienced early treatment failures and fatal outcomes. Depicted are cluster figures of the mRNA expression levels for ERBB1 as well as B-lineage surface receptor CD19 in MLC from from limited-stage DLBCL patients as well as advanced-stage DLBCL patients who died within 2.5 years when mean centered to the corresponding mRNA expression levels in the MLC from DLBCL patients who remained alive after 2.5 years. The cluster figure shows the log_2_-transformed fold-change values (blue represents underexpression, and the red color represents overexpression in MLC from patients experiencing early death). (**A**) At 2.5 years, 41 of 188 limited-stage (Stage I or II) DLBCL patients had died, and 147 remained alive. Limited-stage DLBCL patients who died within 2.5 years exhibited an increased expression of ERBB1 mRNA (but not CD19 mRNA) compared to limited-stage DLBCL patients who remained alive after 2.5 years (Probeset EGFR/ERBB1_1565483_at: 1.34-fold increased expression, *p*-value < 8.1 × 10^−4^; Probeset EGFR/ERBB1_1565484_x_at: 1.32-fold increased expression, *p*-value < 1.4 × 10^−3^). (**B**) At 2.5 years, 89 of 218 advanced-stage (Stage III or IV) DLBCL patients had died, and 129 remained alive. Advanced-stage DLBCL patients who died within 2.5 years exhibited an increased expression of ERBB1 mRNA (but not CD19 mRNA) compared to advanced-stage DLBCL patients who remained alive after 2.5 years (Probeset EGFR/ERBB1_1565483_at: 1.56-fold increased expression, *p*-value < 10^−8^; Probeset an EGFR/ERBB1_1565484_x_at: 1.41-fold increased expression, *p*-value = 9.4 × 10^−8^).

Next, we sought to determine if the expression levels of ERBB1 mRNA at the time of diagnosis were higher in DLBCL patients who did not achieve long-term survival after standard frontline therapy. To this end, we first compared ERBB1 mRNA expression levels in the MLC from DLBCL patients with different histologic subtypes who had died within 2.5 years vs. those who remained alive at 2.5 years. As shown in Figure 2, the MLC from patients who had died exhibited a significantly higher ERBB1 mRNA expression level than the MLC from patients in the same subsets who remained alive at 2.5 years. Very similar results were obtained for DLBCL patients with a limited stage (Stage I–II) as well as advanced stage (Stage III or IV) disease (Figure 3). These results suggested that the augmented ERBB1 expression level may be a prognostic biomarker of MLC from DLBCL patients who may have poor early survival outcomes. 

### 3.2. Expression of ERBB1 mRNA in Lymphoma Cells from DLBCL Patients Is Positively Correlated with mRNA Levels of TF Binding to ERBB1 Promoter Sequences

**Figure 4:**
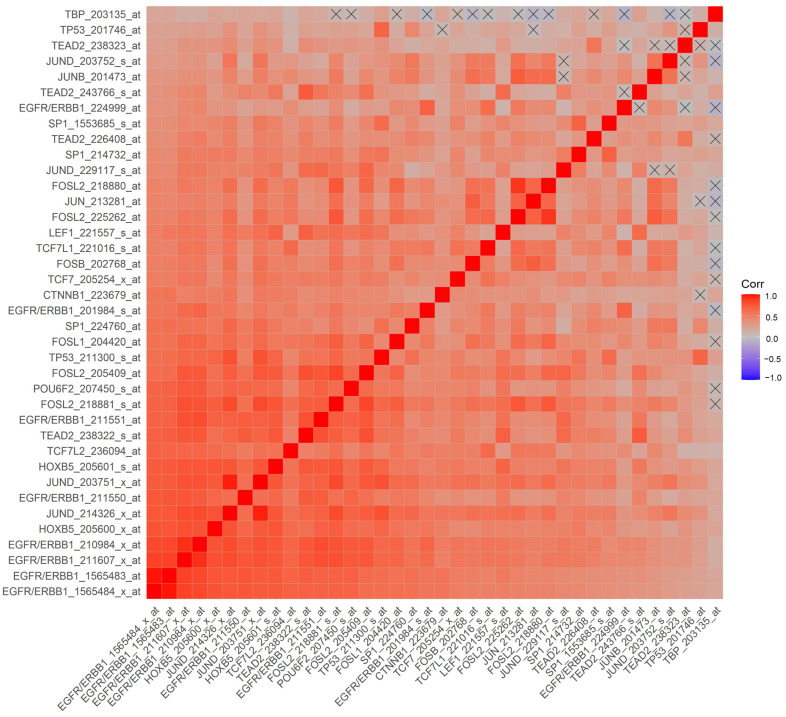
Correlation matrix of mRNA levels for ERBB1 and TF in MLC from DLBCL patients for the probesets with the most significant positive correlations. Pearson correlation coefficients were calculated for MLC from 414 adult DLBCL patients with survival outcome data (GSE10846) (Corr) for ERBB1 mRNA and TF mRNA levels for the indicated probesets (60 probesets representing 21 genes, see Supplementary Materials: Figure S5). Depicted on the heatmap are the most significantly and positively correlated (*p* < 0.0001, FDR < 0.001) mRNA levels according to the specific probesets. Eight out of the nine probesets for ERRB1 were positively correlated with each other, and the probesets represented TF bound to the SP-1 promoter (3 probesets for SP1), ETF promoter (TEAD2, 4 probesets), HOXB5 promoter (HOXB5, 2 probesets), TCF/LEF1 promoter (CTNNB1 and LEF1), TCF7L2 (TCF7L1, TCF7L2, and TCF7), RPF-1 promoter (POU6F2), AP-1 promoter (FOSB, FOSL1, FOSL2 (4 probesets), JUN, JUNB, JUND (4 probesets)) and p53 promoter (TP53; 2 probesets).

**Figure 5:**
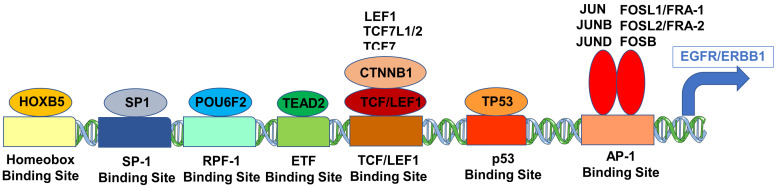
Cartoon figure of the proposed molecular mechanism for the upregulated expression of ERBB1 mRNA in MLC from DLBCL patients. Depicted are TF (oval shapes) that showed the most significant correlations with ERBB1 expression at the mRNA level and their respective binding sites to the ERBB1 gene (rectangles).

We hypothesized that a higher ERBB1 mRNA expression in MLC from DLBCL patients may be owing to higher expression levels of those TF that can transcriptionally activate the ERBB1 gene expression. Pairwise correlations of mRNA levels measured using 60 probesets identified 2738 significant correlations (*p*-values < 0.05; false discovery rate (FDR) = 0.05) (Supplementary Materials: Figure S5). We found that increased mRNA levels for several of these TF were positively correlated with increased ERBB1 mRNA levels (Supplementary Materials: Figure S5, Tables S6 and S7). Particularly, our analyses showed that an increased ERBB1 mRNA expression was correlated with higher mRNA levels for (i) TEAD2 binding to the ETF-binding site, (ii) TF that bound to the AP-1 binding site, (iii) TF that bound to the beta-Catenin TCF/LEF1 binding site, (iv) HOXB5, (v) TF that bound to the SP-1 binding site, (vi) TF that bound to the p53 promoter, (vii) the DNA binding protein IRF1 that bound to the IRF-1 promoter and (viii) the POU6F2 that bound to the RPF-1 promoter site (Supplementary Materials: Figure S5). Highly significant positive correlations (*p* < 0.0001, FDR < 0.001) were observed between the ERBB1 mRNA levels and mRNA levels of the TF and recognized the promoter sites for SP-1, ETF (TEAD2), HOXB5, TCF/LEF1 (CTNNB1 and LEF1), TCF7L2 (TCF7L1, TCF7L2, and TCF7), RPF-1 (POU6F2), AP-1 (FOSB, FOSL1, FOSL2, JUN, JUNB, JUND and p53 (Figure 4 and Figure 5).

### 3.3. Amplified ERBB1 mRNA Level Is Associated with Poor Prognosis in DLBCL 

**Figure 6:**
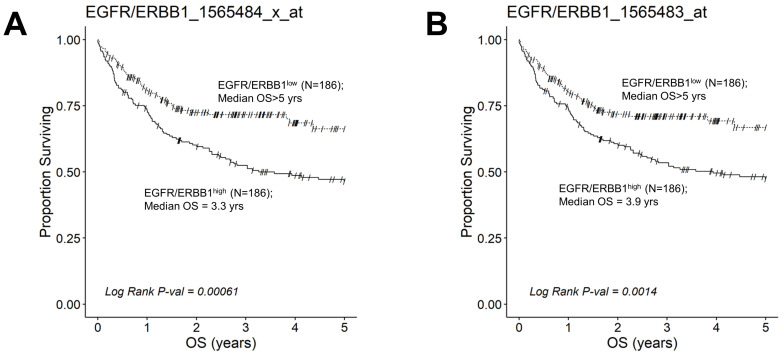
Augmented expression of ERBB1 mRNA is associated with shorter OS in newly diagnosed DLBCL patients. OS data from newly diagnosed DLBCL patients (GSE10846) were combined with mRNA expression data for ERBB1 probesets 1565484_x_at and 1565483_at to assess the potential impact of ERBB1 expression levels on OS. Both ERBB1 expression data and survival data were available for 414 DLBCL patients, who were all included in this analysis. RMA normalized values from 414 newly diagnosed DLBCL patients were rank-ordered according to the expression of ERBB1 mRNA levels and according to 2 specific probesets, namely EGFR/ERBB1_1565484_x_at (Panel **A**) and EGFR/ERBB1_ 1565483_at (Panel **B**). We compared the OS outcome for DLBCL patients with the highest ERBB1 mRNA expression level in their MLC (i.e., top 45% with the highest observed expression level; N = 186) with the OS outcome for DLBCL patients with the lowest expression level of ERBB1 mRNA in their MLC (i.e., bottom 45% with the lowest observed expression level; N = 186). (**A**) Patients with the highest mRNA expression level for the ERBB1 probeset 1565484_x_at in their MLC (EGFR/ERBB1^high^) exhibited a significantly worse OS outcome than those with the lowest ERBB1 mRNA expression level in their MLC (EGFR/ERBB1^low^) (Log-rank Chi-square value = 11.74, *p*-value = 0.00061; Median = 3.26 ((95% CI: 2.31–NA)) years for the high expression group compared to a 66% survival probability at 5 years for the low expression group). (**B**). Likewise, for the ERBB1 probeset 1565483_at, EGFR/ERBB1^high^ patients exhibited a significantly worse OS outcome than EGFR/ERBB1^low^ patients (Log-rank Chi-square value = 10.21, *p*-value = 0.0014; Median = 3.93 ((95% CI: 2.45–NA)) years for the high expression group compared to a 67% survival probability at 5 years for the low expression group).

**Figure 7:**
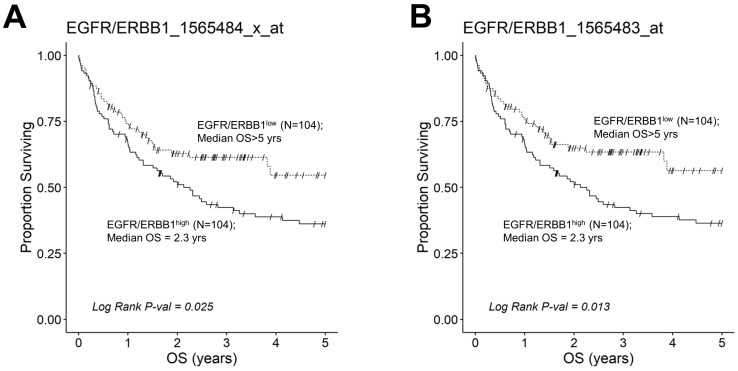
Augmented expression of ERBB1 mRNA is associated with shorter OS in non-GCB (ABC-type + unclassified) type DLBCL patients. OS data from newly diagnosed non-GCB type DLBCL patients (GSE10846; Pooled 167 ABC-type DLBCL patients with 64 unclassified DLBCL patients (Total N =231)) were combined with mRNA expression data for the ERBB1 probesets EGFR/ERBB1_1565484_x_at (Panel **A**) and EGFR/ERBB1_1565483_at (Panel **B**) to assess the potential impact of ERBB1 mRNA expression levels on the OS. We compared the OS outcome for non-GCB DLBCL patients with the highest ERBB1 mRNA expression level in their MLC (i.e., top 45% with the highest observed expression level; N = 104) with the OS outcome for non-GCB DLBCL patients with the lowest expression level of ERBB1 mRNA in their MLC (i.e., bottom 45% with the lowest observed expression level; N = 104). (**A**) Patients with the highest mRNA expression level for the ERBB1 probeset 1565484_x_at in their MLC (EGFR/ERBB1^high^) exhibited a significantly worse OS outcome than those with the lowest ERBB1 mRNA expression level in their MLC (EGFR/ERBB1^low^) (Log-rank Chi-square value = 5.04, *p*-value = 0.025; Median = 2.26 ((95% CI: 1.31–3.58)) years for high the expression group compared to a 54% survival probability at 5yrs for the low expression group). (**B**). Likewise, for the ERBB1 probeset 1565483_at, EGFR/ERBB1^high^ patients exhibited a significantly worse OS outcome than EGFR/ERBB1^low^ patients (Log-rank Chi-square value = 6.14, *p*-value = 0.013; Median = 2.26 ((95% CI: 1.31–3.58)) years for the high expression group compared to >5 years with a 56% survival probability at 5 yrs for the low expression group).

**Figure 8:**
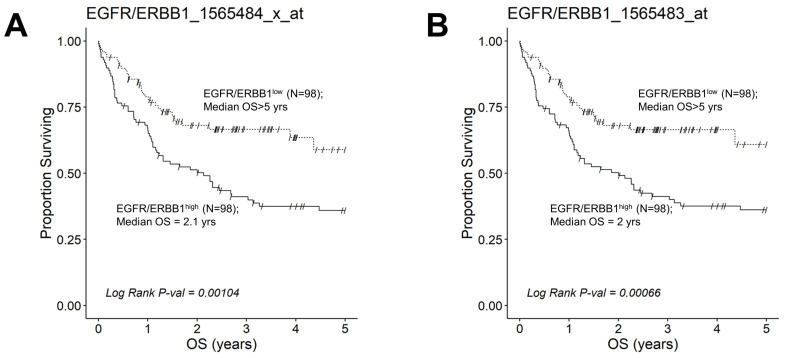
Augmented expression of ERBB1 mRNA is associated with shorter OS in advanced-stage DLBCL patients. OS data from 218 newly diagnosed advanced-stage DLBCL patients (GSE10846; Pooled 97 Stage III patients with 121 Stage IV patients were combined with mRNA expression data for the ERBB1 probesets EGFR/ERBB1_1565484_x_at (Panel **A**) and EGFR/ERBB1_1565483_at (Panel **B**) to assess the potential impact of ERBB1 mRNA expression levels on OS. We compared the OS outcome for advanced-stage DLBCL patients with the highest ERBB1 mRNA expression level in their MLC (i.e., top 45% with the highest observed expression level; N = 98) with OS outcome for advanced-stage DLBCL patients with the lowest expression level of ERBB1 mRNA in their MLC (i.e., bottom 45% with the lowest observed expression level; N = 98). (**A**) Patients with the highest mRNA expression level for the ERBB1 probeset 1565484_x_at in their MLC (EGFR/ERBB1^high^) exhibited a significantly worse OS outcome than those with the lowest ERBB1 mRNA expression level in their MLC (EGFR/ERBB1^low^) (Log-rank Chi-square value = 10.75, *p*-value = 0.00104; 2.13 ((95% CI: 1.16–3.26)) years vs. >5 years, 59% survival probability at 5 yrs for high and low expression groups, respectively). There were 60 deaths in the EGFR/ERBB1^high^ group and 32 events in the EGFR/ERBB1^low^ group. (**B**). Likewise, for the ERBB1 probeset 1565483_at, EGFR/ERBB1^high^ patients exhibited a significantly worse OS outcome than EGFR/ERBB1^low^ patients (Log-rank Chi-square value = 11.61, *p*-value = 0.00066; 2 ((95% CI: 1.1–3.26)) years vs. >5 years with 61% survival probability at 5 yrs for high and low expression groups, respectively). There were 60 deaths in the EGFR/ERBB1^high^ group and 31 events in the EGFR/ERBB1^low^ group.

We next evaluated the effect of amplified MLC ERBB1 mRNA levels on the survival outcome of DLBCL patients. To this end, we compared the OS for DLBCL patients whose MLC expressed high levels of ERBB1 mRNA with the OS times for DLBCL patients whose MLC expressed low levels of ERBB1 mRNA. EGFR/ERBB1^high^ patients whose MLC expressed the highest ERBB1 mRNA levels had a significantly worse OS compared to EGFR/ERBB1^low^ patients with the lowest ERBB1 mRNA expression levels in their MLC (ERBB1 probeset 1565484_x_at: Log-rank Chi-square value = 11.74, *p*-value = 0.00061) (Figure 6). The apparent prognostic significance of amplified ERBB1 expression was confirmed for the high-risk non-GCB (ABC-type + unclassified) type (Figure 7) as well as advanced-stage DLBCL patients (Figure 8). By comparison, limited-stage (Stage I or II) DLBCL patients with the highest level of ERBB1 mRNA expression in MLC showed a statistically insignificant trend toward shorter OS times (Supplementary Materials: Figure S6). 

We next used an independent validation dataset on RNAseq-based mRNA profiles and matched the survival data of 47 DLBCL patients from the TCGA project that were available to further evaluate the prognostic effect of high-level EGFR/ERBB1 mRNA expression. Patients with the highest intra-tumor ERBB1 mRNA expression levels (top 40%, EGFR/ERBB1^high^) had worse DFS (Supplementary Materials: Figure S7A) and OS (Supplementary Materials: Figure S7B) than patients with the lowest intra-tumor ERBB1 mRNA levels (bottom 40%, EGFR/ERBB1^low^). The probability of 100 month-DFS for EGFR/ERBB1^low^ patients was 94% (95%CI = 84–100%), which was above the upper 95% confidence band for the 100 month-DFS of 57% for EGFR/ERBB1^high^ patients (95%CI = 36–92%). Similarly, the probability of 100-month survival was 100% for EGFR/ERBB1^low^ patients, which was above the upper 95% confidence band for the 100 month-survival of 76% for EGFR/ERBB1^high^ patients (95%CI = 59–99%). However, the increased hazard ratios (HR) observed for DFS (HR = 3.6) and OS (HR = 5.4) in the EGFR/ERBB1^high^ patients of this small validation cohort did not reach statistical significance, likely due to the reduced statistical power arising from the broad standard error estimations of DFS and OS outcomes as well as the very small sample size.

## 4. Discussion

DLBCL has an incidence of seven cases per 100,000 people per year [44,45,46,47,48]. This aggressive non-Hodgkin’s lymphoma (NHL) is thought to originate from transformed mature B-cell populations according to a complex molecular pathogenesis and displays a marked biological and prognostic heterogeneity. The majority of newly diagnosed patients show advanced (Stage III or IV) disease requiring intensive immunochemotherapy [49,50]. The GCB-type DLBCL is associated with a more favorable prognosis than the ABC-type DLBCL. Recent therapeutic advances yielded significant improvements in the survival outcomes of patients with B-lineage lymphoid malignancies, including DLBCL [51,52,53,54,55,56,57]. Furthermore, the therapeutic landscape is continually evolving with the introduction of biotherapy platforms, such as monoclonal antibodies, bispecific antibodies, and CAR-T cells [55]. However, drug resistance continues to result in treatment failure [51,52,53,54,55,56,57]. For relapsed or refractory DLBCL patients, salvage treatment strategies include either CD20 or CD79b-directed antibodies or antibody-drug conjugates, CD19-directed antibodies, and CAR-T cells as well as bone marrow or peripheral blood stem cell transplantation [51,52,53,54,55,56,57]. More effective treatment strategies are urgently needed for eradication of refractory MLC clones that contribute to disappointingly poor outcomes for DLBCL patients experiencing a relapse on contemporary frontline regimens.

This study examined the transcript-level expression of ERBB1 in MLC from 498 newly diagnosed DLBCL patients. MLC in each histological DLBCL subset exhibited an amplified ERBB1 expression compared to normal control BLC populations, and the highest expression levels were observed in the non-GCB subset. Furthermore, our analyses revealed significantly higher ERBB1 mRNA expression levels in the MLC from DLBCL patients who died within 2.5 years vs. patients who remained alive after 2.5 years. These results suggested that the augmented ERBB1 mRNA expression level may be a prognostic biomarker for DLBCL patients associated with poor early survival outcomes. Notably, a comparison of DLBCL patients with the highest versus lowest expression levels for ERBB1 mRNA in their MLC revealed a significantly worse OS outcome for the EGFR/ERBB1^high^ group. The prognostic significance of ERBB1 mRNA amplification was confirmed for the high-risk non-GCB (ABC-type + unclassified) type as well as advanced-stage DLBCL patients (Figure 7 and Figure 8). 

We tested the hypothesis that amplified mRNA levels of the ERBB1 in MLC from DLBCL patients may be driven by the increased expression of specific TF that transcriptionally activate ERBB1 expression. Our findings demonstrated that an increased ERBB1 mRNA expression showed statistically significant correlations with upregulated mRNA levels of several such TF, including ETF [23,24,25], SP-1 [26,27,28,29,30], TCF/LEF1 [31,32], HOXB5 [33,34], RPF-1 [35], p53 [36] and AP-1 [22,36,37,38]. We propose a model according to which the observed upregulation of ERBB1 mRNA expression in DLBCL can be driven transcriptionally by a concerted action of several TF (Figure 4 and Figure 5).

Our study has significant limitations, including the absence of a large validation dataset, the bioinformatics-based analyses being used without additional supportive laboratory testing of ERBB1 mRNA levels using other methods (e.g., quantitative RT-PCR, RNA sequencing), and a lack of data to show MLC from EGFR/ERBB1^high^ DLBCL patients with amplified ERBB1 protein expression and PTK activity. Our results need to be verified in a prospective study with integrated RNAseq and biochemical testing in a larger DLBCL patient population. A multivariate analysis of the prognostic significance of ERBB1 overexpression in relationship to other prognostic factors and patient treatment is very important. If confirmed and extended in additional validation and proof of concept studies, our findings suggest that ERBB1 may emerge as a genuine therapeutic target in DLBCL.

Targeting ERBB1 with FDA-approved small molecule ERBB1 inhibitors as well as monoclonal antibodies (Table 1) [5,11] could potentially improve the treatment options for high-risk or relapsed DLBCL patients. Our results presented herein warrant further examination of the therapeutic potential of already FDA-approved small molecule inhibitors as well as monoclonal antibodies targeting ERBB1 that could be repurposed for use in high-risk or relapsed DLBCL. Besides ERBB1, other ErbB family PTK may also affect the biology of malignant lymphomas. A recent study suggested that the overexpression of ERBB4 may render MLC resistant to inhibitors of BTK and PI3K [58]. Our findings should encourage a systematic review of the expression of ErbB family PTK in malignant lymphomas beyond DLBCL and in relation to clinical outcomes. 

## 5. Conclusions

Upregulated ERBB1 expression in DLBCL and its subtypes was associated with significantly worse OS. Our results encourage the further evaluation of the prognostic significance of high-level ERBB1 mRNA expression and the clinical potential of ERBB1-targeting therapeutics as personalized medicines in high-risk DLBCL.

## Figures and Tables

**Table 1:** FDA-approved inhibitors of EGFR/ERBB1.

Target Kinase	Drug	Brand
EGFR	Gefitinib	ZD1839, Iressa
EGFR	Dacomitinib	PF- 00299804, Visimpro
EGFR	Erlotinib	OSI-744, Tarceva
EGFR T790M	Osimertinib	AZD- 9292, Tagrisso
EGFR with exon 20 insertions	Mobocertinib	TAK-788, AP-32788, EXKIVITY
ErbB1/2/4	Afatinib	Gilotrif, Tovok
ErbB1/2/HER2	Lapatinib	Tykerb

## Data Availability

We used the publicly available archived gene expression profiling datasets GSE10846, GSE12195, and GSE12453 generated in the GeneChip Human Genome U133 Plus 2.0 Array platform (ThermoFischer Scientific, Waltham, MA, USA) and downloaded from the NCBI repository (https://www.ncbi.nlm.nih.gov/geo/; accessed on 30 July 2022). We created a working database for the probeset-level expression profiling of primary malignant lymphoma cells isolated from lymph node specimens of newly diagnosed patients with DLBCL (N = 498, GSE10846, GSE12195, GSE12453). In addition, we used the archived dataset from GSE12195 and GSE12453 for normal B-lineage lymphoid cell (BLC) populations for comparison with malignant cells. The original contributions presented in this study are included in the article/Supplementary Material. Further inquiries can be directed to the corresponding author.

## Supplementary Materials

The following supporting information can be downloaded at: https://www.mdpi.com/article/10.3390/jpm13050770/s1, Figure S1: Upregulated Expression of ERBB1 mRNA in MLC from Adult GCB-type DLBCL Patients. Figure S2: Upregulated Expression of ERBB1 mRNA in MLC from Adult ABC-type DLBCL Patients. Figure S3: Upregulated Expression of ERBB1 mRNA in MLC from Adult non-GCB-type (ABC-type plus unclassified) DLBCL Patients. Figure S4: ERBB1 mRNA expression levels in MLC from Adult Non-GCB type versus GCB-type DLBCL Patients. Figure S5: Correlation matrix of mRNA levels for ERBB1 and transcription factors in MLC from DLBCL patients. Figure S6: Augmented Expression of ERBB1 mRNA is Associated with a trend towards shorter OS in Limited Stage DLBCL patients. Figure S7: The unfavorable impact of RNAseq-based high intra-tumor ERBB1 mRNA expression on disease-free survival (DFS) and overall survival (OS) outcomes of DLBCL Patients; Supplementary Materials: Table S1: mRNA levels of ErbB family protein tyrosine kinases in MLC from DLBCL patients versus normal BLC. Supplementary Materials: Table S2: mRNA levels of ErbB family protein tyrosine kinases in MLC from GCB-type DLBCL patients vs. normal BLC. Supplementary Materials: Table S3: mRNA levels of ErbB family protein tyrosine kinases in MLC from ABC-type DLBCL patients vs. normal BLC. Supplementary Materials: Table S4: mRNA levels of ErbB family protein tyrosine kinases in MLC from non-GCB-type DLBCL patients vs. normal BLC. Supplementary Materials: Table S5: mRNA levels of ErbB family protein tyrosine kinases in MLC from non-GCB vs. GCB-type -DLBCL patients. Supplementary Materials: Table S6: Correlated expression of mRNA for ERBB1 and specific transcription factors in MLC from DLBCL patients: Data for the Probeset EGFR/ERBB1_1565483_at. Supplementary Materials: Table S7: Correlated expression of mRNA for ERBB1 and specific transcription factors in MLC from DLBCL patients: Data for the Probeset EGFR/ERBB1_1565484_at.
