# Supplementary material for: Upregulated Expression of ErbB1 in Diffuse Large B-Cell Lymphoma as a Predictor of Poor Overall Survival Outcome
Source: J Pers Med. 2023 Apr 29;13(5):770. doi: 10.3390/jpm13050770 (PMC10221820; doi:10.3390/jpm13050770)
Supplement: Supplementary file 1 [file jpm-13-00770-s001.zip › jpm-2313641-supplementary.pdf]

Journal of
Personalized
Medicine

MDPI

Supplemental Figures and Tables

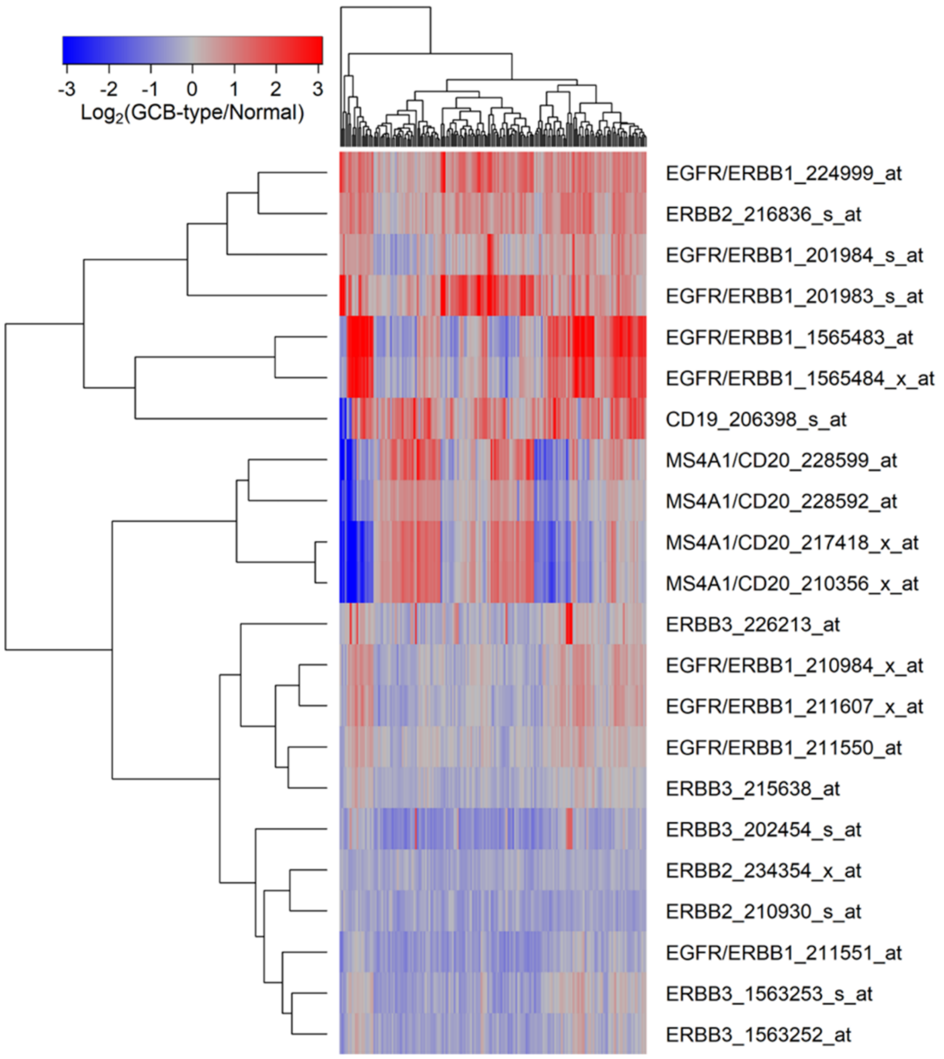

Figure S1. Upregulated Expression of ERBB1 mRNA in MLC from Adult GCB-type DLBCL Patients. Depicted is a cluster figure of the mRNA expression levels for ERBB1, ERBB2, ERBB3 as well as B-lineage surface receptors CD19 and CD20 in MLC from GCB-type DLBCL patients (N=183) mean centered to the corresponding mRNA expression levels in normal BLC (N=45). The cluster figure shows the log_2-transformed fold-change values (blue represents underexpression and red color represents overexpression in samples from GCB-type DLBCL patients). ERBB1 mRNA was significantly upregulated (Probeset ERBB1_201983_s_at : Fold Change = 2.01; P-value < 10^{-8}; Probeset ERBB1_224999_at: Fold Change = 1.86; P-value < 10^{-8}; Probeset ERBB1_1565483_at : Fold Change = 1.74; P-value < 10^{-8}) (**Table S2**).

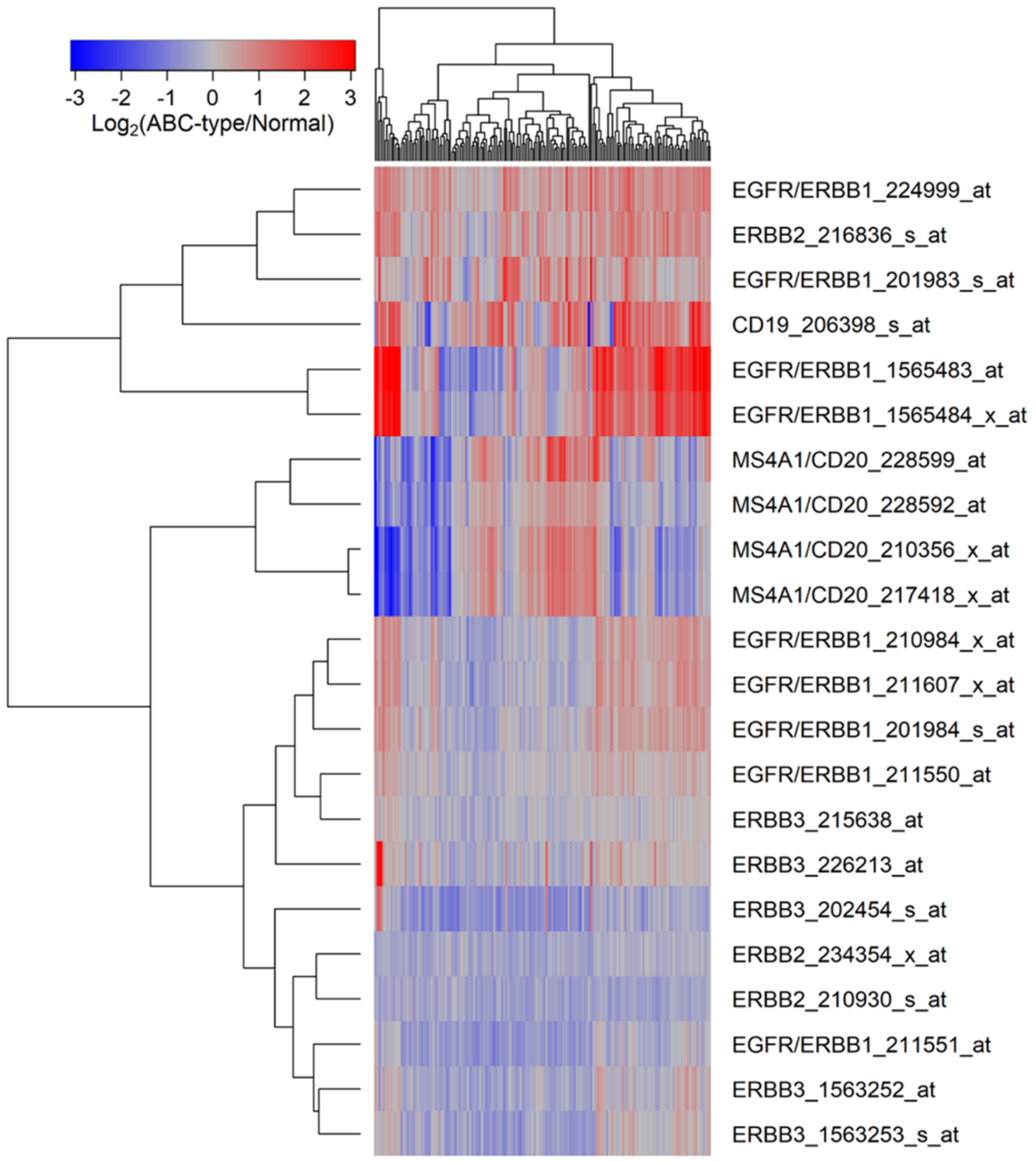

Figure S2. Upregulated Expression of ERBB1 mRNA in MLC from Adult ABC-type DLBCL Patients. Depicted is a cluster figure of the mRNA expression levels for ERBB1, ERBB2, ERBB3 as well as B-lineage surface receptors CD19 and CD20 in MLC from ABC-type DLBCL patients (N=167) mean centered to the corresponding mRNA expression levels in normal BLC (N=45). The cluster figure shows the log_2-transformed fold-change values (blue represents underexpression and red color represents overexpression in samples from ABC-type DLBCL patients). ERBB1 mRNA was significantly upregulated (Probeset ERBB1_1565483_at: Fold Change = 1.84; P-value < 10^{-8}; Probeset ERBB1_1565484_x_at : Fold Change = 1.76; P-value < 10^{-8}). CD19 mRNA was also upregulated (Probeset CD19_206398_s_at : Fold Change = 1.6; P-value = 1.3 x10^{-7}) (**Table S3**).

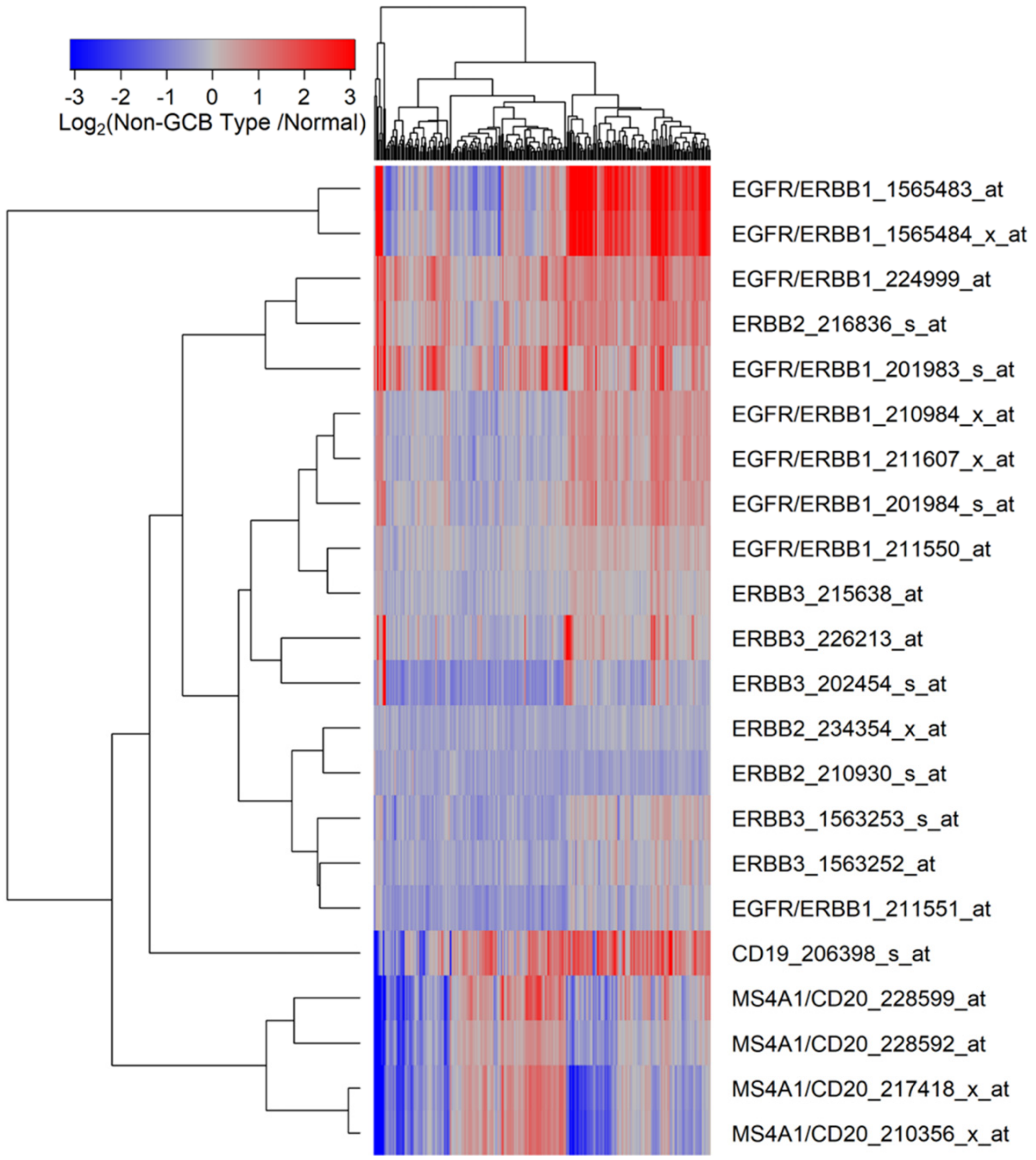

Figure S3. Upregulated Expression of ERBB1 mRNA in MLC from Adult non-GCB-type (ABC-type plus unclassified) DLBCL Patients. Depicted is a cluster figure of the mRNA expression levels for ERBB1, ERBB2, ERBB3 as well as B-lineage surface receptors CD19 and CD20 in MLC from non-GCB-type DLBCL patients (N=231) mean centered to the corresponding mRNA expression levels in normal BLC (N=45). The cluster figure shows the log_2-transformed fold-change values (blue represents underexpression and red color represents overexpression in samples from non-GCB-type DLBCL patients). ERBB1 mRNA was significantly upregulated (Probeset ERBB1_1565483_at: Fold Change = 2.06; P-value < 10^{-8}; Probeset ERBB1_1565484_x_at : Fold Change = 1.97; P-value < 10^{-8}; Probeset ERBB1_224999_at: Fold Change = 1.73; P-value = 2.4 x10^{-8}). (**Table S4)**

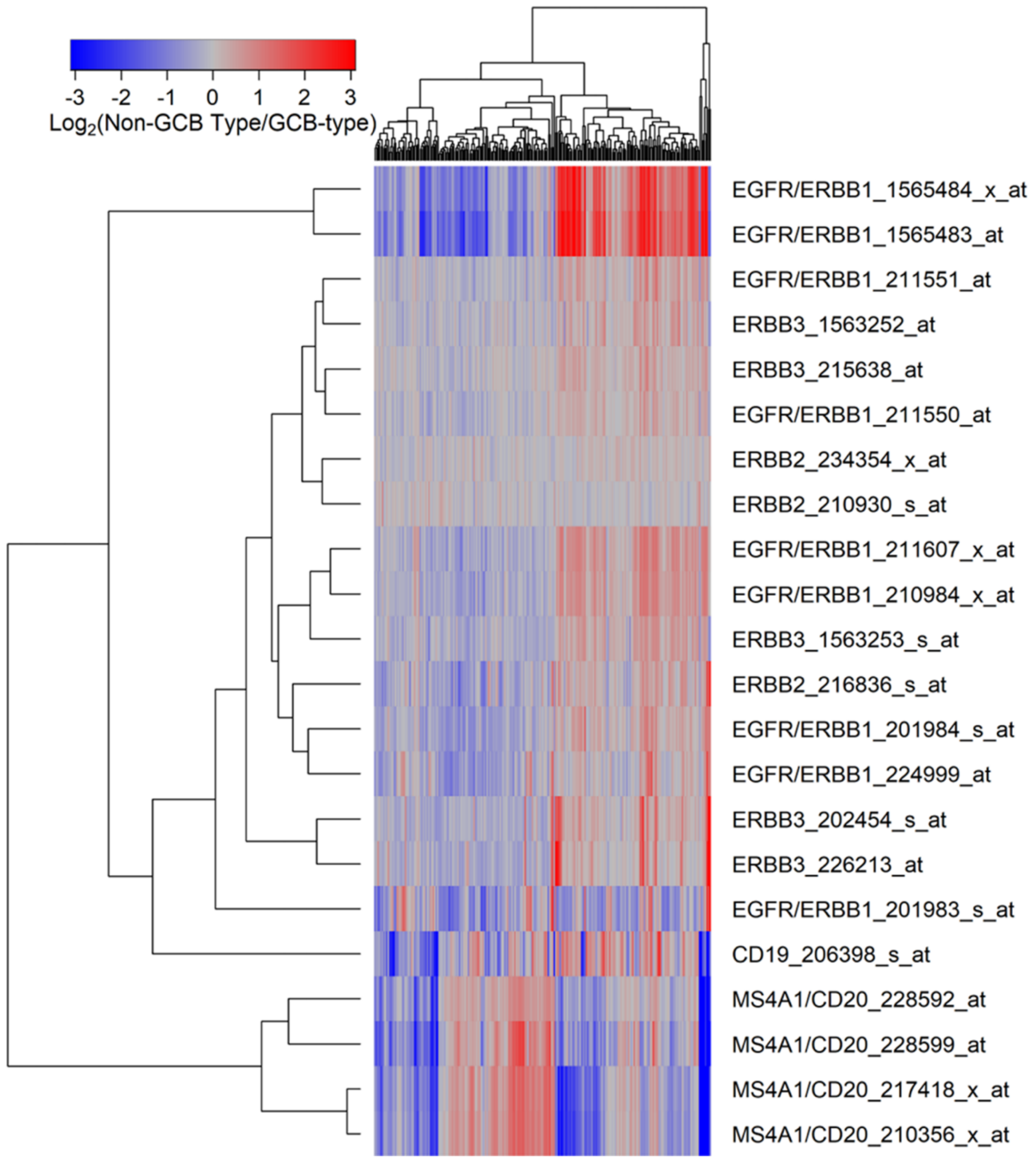

Figure S4. ERBB1 mRNA expression levels in MLC from Adult Non-GCB type versus GCB-type DLBCL Patients.

Depicted is a cluster figure of the mRNA expression levels for ERBB1, ERBB2, ERBB3 as well as B-lineage surface receptors CD19 and CD20 in MLC from non-GCB-type DLBCL patients (N=231) mean centered to the corresponding mRNA expression levels in MLC from GCB-type (N=183). The cluster figure shows the $\log_2$-transformed fold-change values (blue represents underexpression and red color represents overexpression in samples from non-GCB-type DLBCL patients). ERBB1 mRNA was significantly upregulated for 2 probesets (Probeset ERBB1_1565484_x_at: Fold Change = 1.21; P-value = $9.8\ 10^{-4}$; Probeset ERBB1_1565483_at (Fold Change = 1.18; P-value = 0.0032) (**Table S5**).

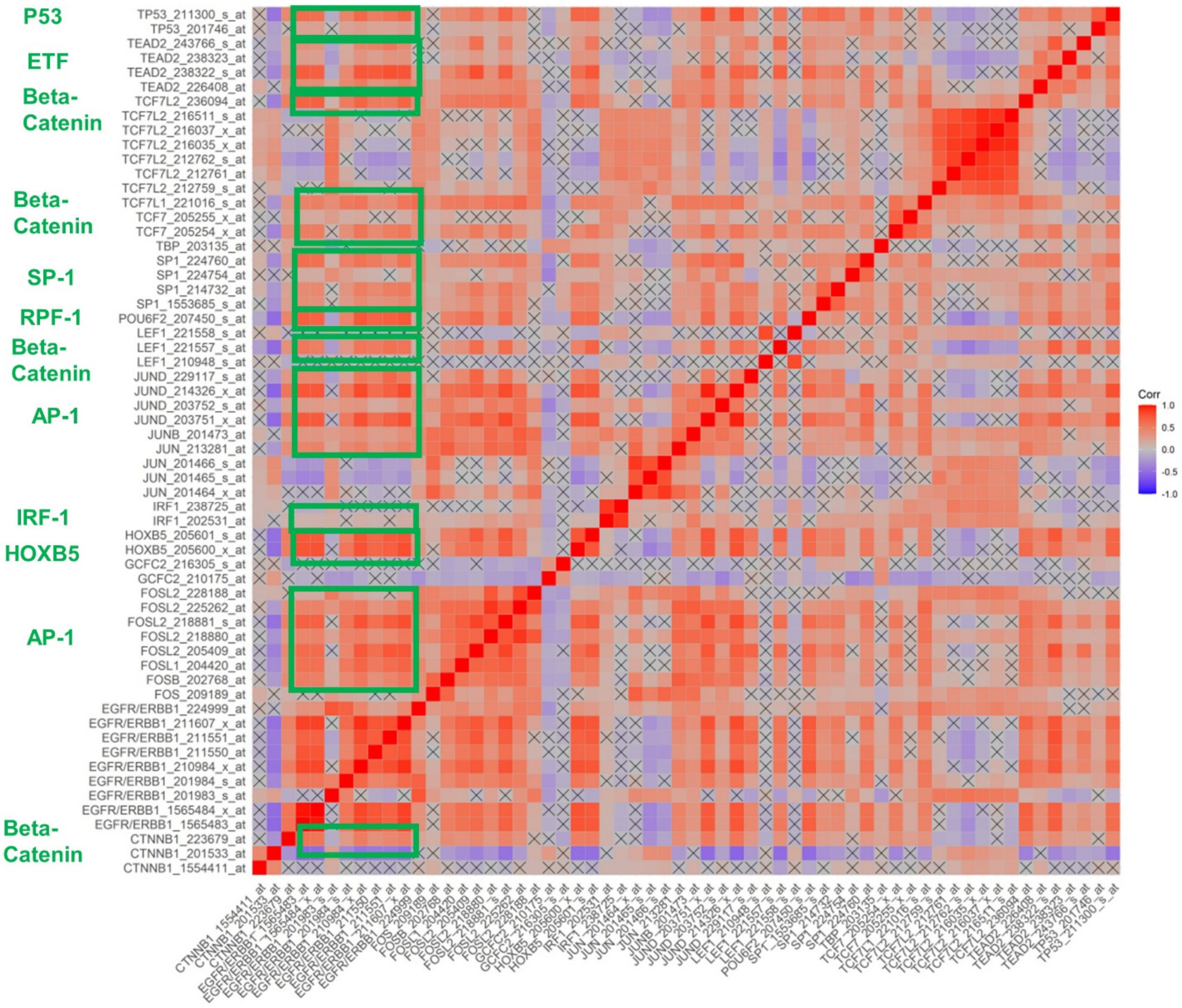

Figure S5. Correlation matrix of mRNA levels for ERBB1 and transcription factors in MLC from DLBCL patients.

Pearson correlation coefficients were calculated for MLC from 414 adult DLBCL patients with survival outcome data (GSE10846) (Corr) for ERBB1 mRNA and TF mRNA levels for the indicated probesets (60 probesets representing 21 genes. Depicted on the heatmap are the color coded correlation coefficients ranging from positive (red) to negative correlations (blue) clustered according to similarly expressed probesets. A total of 3540 pairwise correlations were performed (excluding 60 self correlations), of which 2738 were deemed to be significant ($P<0.05$ and FDR = 0.05; non-significant correlations are indicated with a black cross in the heat map). Eight probesets for ERRB1 formed a highly co-regulated cluster. The dark green boxes show co-regulated probesets for transcription factors that bind to the promoter regions labeled with dark green text that were positively correlated with the 8 ERBB1 probesets. Four probesets were for mRNA of the TF TEAD2 that binds to the ETF promoter; 13 probesets were for mRNA of TF that bind to the AP-1 promoter; 6 probesets were for TF that bind to the Beta-Catenin TCF promoter; 2 probesets for mRNA of TF that bind the HOXB5 promoter; 4 probesets for TF that bind to the SP-1 promoter; 2 probesets for TF that bind the p53 promoter, 1 probeset each for mRNA of POU6F2 that binds to the RPF-1 promoter and IRF1 that binds to the IRF-1 promoter were positively correlated with ERRB1 mRNA expression.

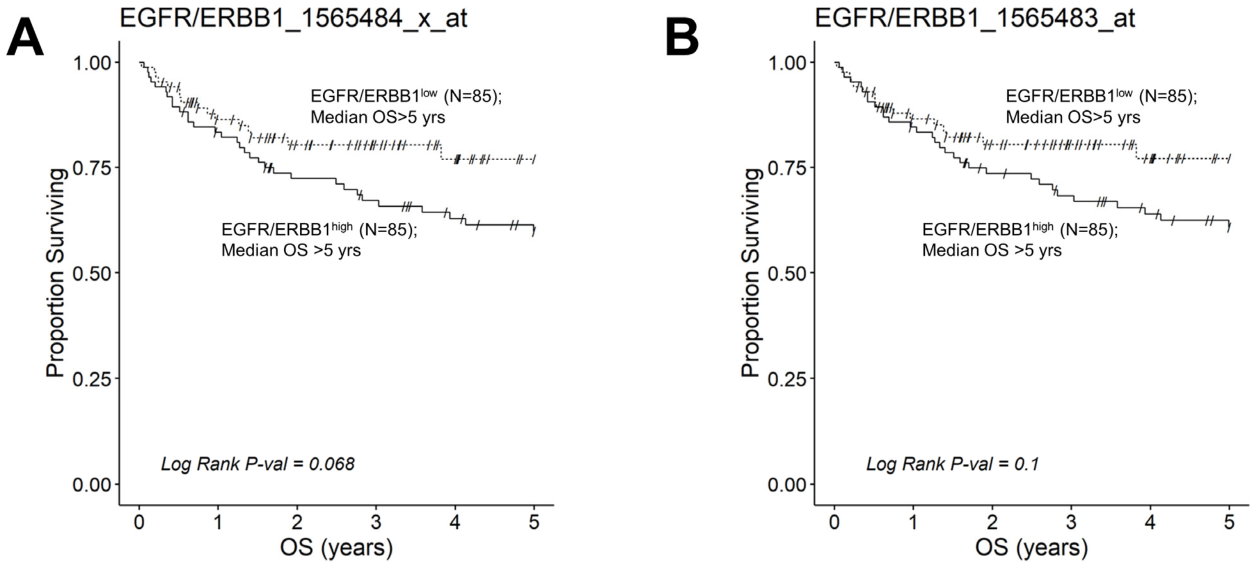

Figure S6. Augmented Expression of ERBB1 mRNA is Associated with a trend towards shorter OS in Limited Stage DLBCL patients

OS data from 188 newly diagnosed limited stage DLBCL patients (GSE10846; Pooled 66 Stage I patients and 122 Stage II patients was combined with mRNA expression data for the ERBB1 probesets EGFR/ERBB1_1565484_x_at (Panel A) and EGFR/ERBB1_1565483_at (Panel B) to assess the potential impact of ERBB1 mRNA expression levels on the OS. We compared the OS outcome for advanced stage DLBCL patients with the highest ERBB1 mRNA expression level in their MLC (i.e., top 45% with the highest observed expression level; N=85) with the OS outcome for advanced stage DLBCL patients with the lowest expression level of ERBB1 mRNA in their MLC (i.e., bottom 45% with the lowest observed expression level; N=85). [A] Patients with the highest mRNA expression level for the ERBB1 probeset 1565484_x_at in their MLC (EGFR/ERBB1^{high}) exhibited a statistically insignificant worse OS outcome than those with the lowest ERBB1 mRNA expression level in their MLC (EGFR/ERBB1^{low}) (Log-rank Chi-square value = 3.34, P-value = 0.068; 60% vs. 77% survival probability at 5yrs for high and low expression groups respectively). There were 32 deaths in the EGFR/ERBB1^{high} group and 16 events in the EGFR/ERBB1^{low} group. [B]. Likewise, for the ERBB1 probeset 1565483_at, EGFR/ERBB1^{high} patients exhibited a statistically insignificant worse OS outcome than EGFR/ERBB1^{low} patients (Log-rank Chi-square value = 2.71, P-value = 0.1; 61% vs. 77% survival probability at 5yrs for high and low expression groups respectively). There were 31 deaths in the EGFR/ERBB1^{high} group and 16 events in the EGFR/ERBB1^{low} group.

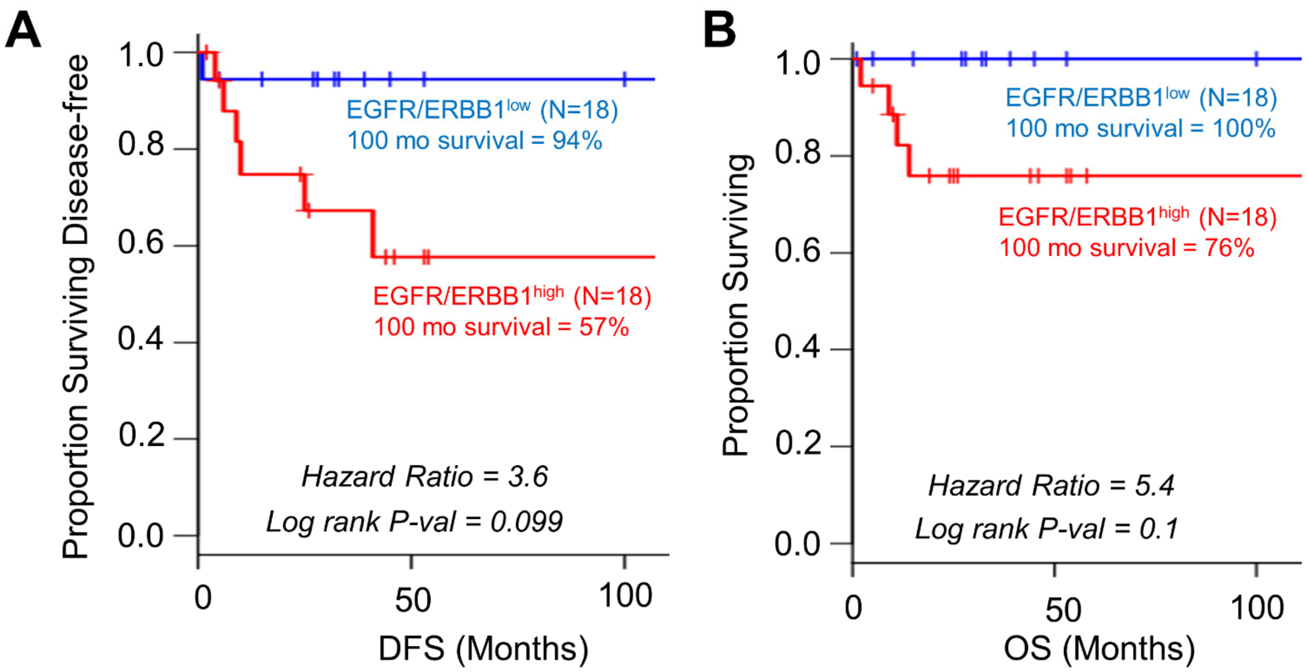

Figure S7. The unfavorable impact of RNAseq-based high intra-tumor ERBB1 mRNA expression on Disease-free survival (DFS) and overall survival (OS) outcomes of DLBCL Patients. Both RNAseq-based ERBB1 mRNA expression data and OS/DFS information were available for 47 DLBCL patients who were all included in this validation cohort from the TCGA data repository. TPM normalized values for ERBB1 mRNA expression levels of MLC were rank ordered to compare the DFS/OS outcomes for DLBCL patients with the highest levels of ERBB1 mRNA expression (EGFR/ERBB1^{high}) in their MLC (viz.: top 40% with the highest observed expression level; N=18) with the DFS/OS outcome for DLBCL patients with the lowest expression level of EGFR/ERBB1 mRNA (EGFR/ERBB1^{low}) in their MLC (i.e., bottom 40% with the lowest observed expression level; N=18). [A] EGFR/ERBB1^{high} patients exhibited worse DFS outcomes and the probability of DFS after 100 months for EGFR/ERBB1^{low} patients was 94% (95%CI = 84 -100%) which was above the upper 95% confidence band for the DFS for EGFR/ERBB1^{high} patients (100-month survival = 57%, 95%CI = 36 – 92%). [B] Similarly, the probability of 100-month survival was 100% for ERBB1^{low} patients which was above the upper 95% confidence band for the 100 month-survival of 76% for ERBB1^{high} patients (95%CI = 59-99%). Increased hazard ratios observed for DFS (HR=3.6) and OS (HR=5.4) in patients with high intra-tumor levels of EGFR/ERBB1 mRNA did not reach statistical significance, this was likely due to reduced power to detect a statistical difference between EGFR/ERBB1^{low} and EGFR/ERBB1^{high} groups of patients arising from the broad standard error estimations of DFS/OS outcomes and small sample size.

Supplemental tables

Table S1. mRNA levels of ErbB family protein tyrosine kinases in MLC from DLBCL patients versus normal BLC

Probeset	Fold Change (DLBCL$^+$/Normal)	Linear Contrast (P-value)
EGFR/ERBB1_201983_s_at	1.94	<1 × 10^{-8}
EGFR/ERBB1_224999_at	1.74	<1 × 10^{-8}
EGFR/ERBB1_1565483_at	1.72	<1 × 10^{-8}
EGFR/ERBB1_1565484_x_at	1.64	7.7 × 10^{-8}
CD19_206398_s_at	1.54	2.5 × 10^{-6}
ERBB2_216836_s_at	1.53	4.1 × 10^{-6}
EGFR/ERBB1_201984_s_at	1.19	5.4 × 10^{-2}
EGFR/ERBB1_210984_x_at	1.09	3.3 × 10^{-1}
EGFR/ERBB1_211607_x_at	1.07	4.3 × 10^{-1}
ERBB3_226213_at	1.05	6.0 × 10^{-1}
EGFR/ERBB1_211550_at	1.03	7.2 × 10^{-1}
ERBB3_215638_at	0.94	5.2 × 10^{-1}
MS4A1/CD20_228599_at	0.93	4.5 × 10^{-1}
ERBB3_1563253_s_at	0.85	8.9 × 10^{-2}
MS4A1/CD20_228592_at	0.85	7.5 × 10^{-2}
MS4A1/CD20_217418_x_at	0.83	5.0 × 10^{-2}
MS4A1/CD20_210356_x_at	0.83	4.6 × 10^{-2}
ERBB3_1563252_at	0.81	2.6 × 10^{-2}
ERBB2_234354_x_at	0.79	1.1 × 10^{-2}
EGFR/ERBB1_211551_at	0.73	8.0 × 10^{-4}
ERBB2_210930_s_at	0.73	7.1 × 10^{-4}
ERBB3_202454_s_at	0.71	1.6 × 10^{-4}

Table S2. **mRNA levels of ErbB family protein tyrosine kinases in MLC from GCB-type DLBCL patients vs. normal BLC**

Probeset	Fold Change (GCB-type DLBCL/Normal)	Linear Contrast (P-value)
EGFR/ERBB1_201983_s_at	2.01	$<1 \times 10^{-8}$
EGFR/ERBB1_224999_at	1.86	$<1 \times 10^{-8}$
EGFR/ERBB1_1565483_at	1.74	$<1 \times 10^{-8}$
CD19_206398_s_at	1.73	$<1 \times 10^{-8}$
EGFR/ERBB1_1565484_x_at	1.64	1.8×10^{-7}
ERBB2_216836_s_at	1.57	1.5×10^{-6}
EGFR/ERBB1_201984_s_at	1.23	2.7×10^{-2}
MS4A1/CD20_228599_at	1.14	1.7×10^{-1}
EGFR/ERBB1_210984_x_at	1.04	6.7×10^{-1}
ERBB3_226213_at	1.04	6.8×10^{-1}
EGFR/ERBB1_211550_at	1.03	7.7×10^{-1}
EGFR/ERBB1_211607_x_at	1.02	8.5×10^{-1}
MS4A1/CD20_217418_x_at	0.97	7.3×10^{-1}
MS4A1/CD20_228592_at	0.97	7.1×10^{-1}
MS4A1/CD20_210356_x_at	0.96	7×10^{-1}
ERBB3_215638_at	0.9	2.5×10^{-1}
ERBB3_1563253_s_at	0.84	6.6×10^{-2}
ERBB3_1563252_at	0.81	2.5×10^{-2}
ERBB2_234354_x_at	0.78	7.6×10^{-3}
ERBB2_210930_s_at	0.72	5.4×10^{-4}
EGFR/ERBB1_211551_at	0.71	2.7×10^{-4}
ERBB3_202454_s_at	0.69	1×10^{-4}

Table S3. mRNA levels of ErbB family protein tyrosine kinases in MLC from ABC-type DLBCL patients vs. normal BLC

Probeset	Fold Change (ABC-type DLBCL/Normal)	Linear Contrast (P-value)
EGFR/ERBB1_1565483_at	1.84	$<1 \times 10^{-8}$
EGFR/ERBB1_1565484_x_at	1.76	$<1 \times 10^{-8}$
CD19_206398_s_at	1.60	1.3×10^{-7}
EGFR/ERBB1_224999_at	1.56	4.3×10^{-7}
ERBB2_216836_s_at	1.44	3.6×10^{-5}
EGFR/ERBB1_201983_s_at	1.38	2.5×10^{-4}
EGFR/ERBB1_201984_s_at	1.10	2.7×10^{-1}
EGFR/ERBB1_210984_x_at	1.08	3.9×10^{-1}
EGFR/ERBB1_211607_x_at	1.07	4.4×10^{-1}
EGFR/ERBB1_211550_at	1.04	6.4×10^{-1}
ERBB3_226213_at	1.03	7.5×10^{-1}
MS4A1/CD20_228599_at	1.02	8×10^{-1}
ERBB3_215638_at	0.94	5.2×10^{-1}
MS4A1/CD20_228592_at	0.89	1.9×10^{-1}
ERBB3_1563252_at	0.87	1.2×10^{-1}
MS4A1/CD20_210356_x_at	0.86	9.6×10^{-2}
MS4A1/CD20_217418_x_at	0.86	9.2×10^{-2}
ERBB3_1563253_s_at	0.85	6.7×10^{-2}
ERBB2_234354_x_at	0.8	1.1×10^{-2}
EGFR/ERBB1_211551_at	0.74	7.9×10^{-4}
ERBB2_210930_s_at	0.74	6.8×10^{-4}
ERBB3_202454_s_at	0.69	3.6×10^{-5}

Table S4. **mRNA levels of ErbB family protein tyrosine kinases in MLC from non-GCB-type DLBCL patients vs. normal BLC**

Probeset	Fold Change (non-GCB type/Normal)	Linear Contrast (P-value)
EGFR/ERBB1_1565483_at	2.06	<1 × 10^{-8}
EGFR/ERBB1_1565484_x_at	1.97	<1 × 10^{-8}
EGFR/ERBB1_224999_at	1.73	2.4 × 10^{-8}
EGFR/ERBB1_201983_s_at	1.61	9.9 × 10^{-7}
CD19_206398_s_at	1.52	2 × 10^{-5}
ERBB2_216836_s_at	1.51	2.4 × 10^{-5}
EGFR/ERBB1_201984_s_at	1.18	9.7 × 10^{-2}
EGFR/ERBB1_210984_x_at	1.12	2.3 × 10^{-1}
EGFR/ERBB1_211607_x_at	1.12	2.5 × 10^{-1}
ERBB3_226213_at	1.10	3.5 × 10^{-1}
EGFR/ERBB1_211550_at	1.05	6.4 × 10^{-1}
ERBB3_215638_at	0.97	7.2 × 10^{-1}
MS4A1/CD20_228599_at	0.90	2.7 × 10^{-1}
ERBB3_1563253_s_at	0.86	1.3 × 10^{-1}
ERBB3_1563252_at	0.85	1 × 10^{-1}
MS4A1/CD20_228592_at	0.80	2.4 × 10^{-2}
ERBB2_234354_x_at	0.79	1.8 × 10^{-2}
EGFR/ERBB1_211551_at	0.75	3.1 × 10^{-3}
ERBB3_202454_s_at	0.75	2.7 × 10^{-3}
MS4A1/CD20_217418_x_at	0.75	2.6 × 10^{-3}
MS4A1/CD20_210356_x_at	0.74	2.2 × 10^{-3}
ERBB2_210930_s_at	0.73	1.3 × 10^{-3}

Table S5. mRNA levels of ErbB family protein tyrosine kinases in MLC from non-GCB vs. GCB-type -DLBCL patients

Probeset	Fold Change (non-GCB type/GCB-type)	Linear Contrast (P-value)
EGFR/ERBB1_1565484_x_at	1.21	9.8×10^{-4}
EGFR/ERBB1_1565483_at	1.18	3.3×10^{-3}
EGFR/ERBB1_211607_x_at	1.10	9.9×10^{-2}
EGFR/ERBB1_210984_x_at	1.08	1.8×10^{-1}
ERBB3_202454_s_at	1.08	1.9×10^{-1}
ERBB3_215638_at	1.08	1.9×10^{-1}
EGFR/ERBB1_211551_at	1.06	3.4×10^{-1}
ERBB3_226213_at	1.06	3.4×10^{-1}
ERBB3_1563252_at	1.05	3.6×10^{-1}
ERBB3_1563253_s_at	1.03	6.6×10^{-1}
ERBB2_234354_x_at	1.02	7.1×10^{-1}
EGFR/ERBB1_211550_at	1.02	7.6×10^{-1}
ERBB2_210930_s_at	1.01	8.3×10^{-1}
ERBB2_216836_s_at	0.96	4.8×10^{-1}
EGFR/ERBB1_201984_s_at	0.95	4.1×10^{-1}
EGFR/ERBB1_224999_at	0.93	2.1×10^{-1}
CD19_206398_s_at	0.88	1.9×10^{-2}
MS4A1/CD20_228592_at	0.83	9.6×10^{-4}
EGFR/ERBB1_201983_s_at	0.80	9.4×10^{-5}
MS4A1/CD20_228599_at	0.79	2.8×10^{-5}
MS4A1/CD20_217418_x_at	0.77	3.8×10^{-6}
MS4A1/CD20_210356_x_at	0.77	3.4×10^{-6}

Table S6. Correlated expression of mRNA for ERBB1 and specific transcription factors in MLC from DLBCL patients: Data for the Probeset EGFR/ERBB1_1565483_at

TF	Correlation Coefficient versus EGFR/ERBB1_1565483_at	P−value
CTNNB1_1554411_at	−0.09	5.7×10^{-2}
CTNNB1_201533_at	−0.52	8.8×10^{-30}
CTNNB1_223679_at	0.58	1.1×10^{-38}
EGFR/ERBB1_1565483_at	1	
EGFR/ERBB1_1565484_x_at	0.98	1.5×10^{-303}
EGFR/ERBB1_201983_s_at	−0.09	8.1×10^{-2}
EGFR/ERBB1_201984_s_at	0.57	9.5×10^{-37}
EGFR/ERBB1_210984_x_at	0.83	3.2×10^{-106}
EGFR/ERBB1_211550_at	0.76	1.2×10^{-80}
EGFR/ERBB1_211551_at	0.69	3.1×10^{-60}
EGFR/ERBB1_211607_x_at	0.85	3.8×10^{-115}
EGFR/ERBB1_224999_at	0.36	7.7×10^{-14}
FOS_209189_at	0.06	2×10^{-1}
FOSB_202768_at	0.51	3.2×10^{-28}
FOSL1_204420_at	0.59	6.7×10^{-41}
FOSL2_205409_at	0.63	7.9×10^{-47}
FOSL2_218880_at	0.46	6.7×10^{-23}
FOSL2_218881_s_at	0.69	1.9×10^{-60}
FOSL2_225262_at	0.47	3.5×10^{-24}
FOSL2_228188_at	0.16	1.3×10^{-3}
GCFC2_210175_at	−0.1	4.1×10^{-2}
GCFC2_216305_s_at	−0.06	2×10^{-1}
HOXB5_205600_x_at	0.76	9.7×10^{-81}
HOXB5_205601_s_at	0.74	2×10^{-72}
IRF1_202531_at	0.1	3.3×10^{-2}
IRF1_238725_at	−0.11	2.5×10^{-2}
JUN_201464_x_at	−0.09	7.7×10^{-2}
JUN_201465_s_at	−0.36	2×10^{-14}
JUN_201466_s_at	−0.27	2.1×10^{-8}
JUN_213281_at	0.46	2.1×10^{-23}
JUNB_201473_at	0.33	8.4×10^{-12}
JUND_203751_x_at	0.73	7.3×10^{-70}
JUND_203752_s_at	0.29	1.6×10^{-9}
JUND_214326_x_at	0.75	2.2×10^{-76}
JUND_229117_s_at	0.43	1.3×10^{-19}
LEF1_210948_s_at	−0.09	8×10^{-2}
LEF1_221557_s_at	0.54	3.3×10^{-33}
LEF1_221558_s_at	−0.09	6.3×10^{-2}
POU6F2_207450_s_at	0.7	5.4×10^{-62}

SP1_1553685_s_at	0.39	9.8×10^{-17}
SP1_214732_at	0.41	3.8×10^{-18}
SP1_224754_at	0.17	5.5×10^{-4}
SP1_224760_at	0.61	7×10^{-44}
TBP_203135_at	0.2	3.4×10^{-5}
TCF7_205254_x_at	0.51	4×10^{-29}
TCF7_205255_x_at	0.18	3.4×10^{-4}
TCF7L1_221016_s_at	0.47	1.2×10^{-24}
TCF7L2_212759_s_at	0.02	7.5×10^{-1}
TCF7L2_212761_at	−0.15	1.7×10^{-3}
TCF7L2_212762_s_at	−0.3	4×10^{-10}
TCF7L2_216035_x_at	−0.12	1.5×10^{-2}
TCF7L2_216037_x_at	0.03	5.3×10^{-1}
TCF7L2_216511_s_at	−0.13	7.3×10^{-3}
TCF7L2_236094_at	0.68	1.3×10^{-58}
TEAD2_226408_at	0.4	3×10^{-17}
TEAD2_238322_s_at	0.71	5×10^{-66}
TEAD2_238323_at	0.27	1.9×10^{-8}
TEAD2_243766_s_at	0.38	5.9×10^{-16}
TP53_201746_at	0.24	5.8×10^{-7}
TP53_211300_s_at	0.62	6.6×10^{-45}

Table S7. Correlated expression of mRNA for ERBB1 and specific transcription factors in MLC from DLBCL patients: Data for the Probeset EGFR/ERBB1_1565484_at

TF	Correlation Coefficient versus EGFR/ERBB1_1565484_x_at	P−value
CTNNB1_1554411_at	-0.08	1.2×10^{-1}
CTNNB1_201533_at	-0.5	2.1×10^{-27}
CTNNB1_223679_at	0.57	4.1×10^{-37}
EGFR/ERBB1_1565483_at	0.98	1.5×10^{-303}
EGFR/ERBB1_1565484_x_at	1	
EGFR/ERBB1_201983_s_at	-0.07	1.6×10^{-1}
EGFR/ERBB1_201984_s_at	0.57	8.9×10^{-38}
EGFR/ERBB1_210984_x_at	0.83	3.7×10^{-106}
EGFR/ERBB1_211550_at	0.75	1.2×10^{-74}
EGFR/ERBB1_211551_at	0.69	2.8×10^{-60}
EGFR/ERBB1_211607_x_at	0.85	3×10^{-116}
EGFR/ERBB1_224999_at	0.37	3.1×10^{-15}
FOS_209189_at	0.07	1.8×10^{-1}
FOSB_202768_at	0.5	7.7×10^{-28}
FOSL1_204420_at	0.6	1.1×10^{-41}
FOSL2_205409_at	0.62	4.6×10^{-46}
FOSL2_218880_at	0.47	1.3×10^{-24}
FOSL2_218881_s_at	0.69	1.1×10^{-59}
FOSL2_225262_at	0.48	8.8×10^{-25}
FOSL2_228188_at	0.17	4×10^{-4}
GCFC2_210175_at	-0.08	1.2×10^{-1}
GCFC2_216305_s_at	-0.05	3.4×10^{-1}
HOXB5_205600_x_at	0.77	2.6×10^{-81}
HOXB5_205601_s_at	0.73	3.3×10^{-69}
IRF1_202531_at	0.11	3.1×10^{-2}
IRF1_238725_at	-0.1	4.3×10^{-2}
JUN_201464_x_at	-0.07	1.4×10^{-1}
JUN_201465_s_at	-0.35	3.4×10^{-13}
JUN_201466_s_at	-0.24	4.9×10^{-7}
JUN_213281_at	0.47	1.2×10^{-24}
JUNB_201473_at	0.33	4.2×10^{-12}
JUND_203751_x_at	0.73	3.8×10^{-70}
JUND_203752_s_at	0.3	6.5×10^{-10}
JUND_214326_x_at	0.75	1.6×10^{-75}
JUND_229117_s_at	0.44	7.5×10^{-21}
LEF1_210948_s_at	-0.09	5.8×10^{-2}
LEF1_221557_s_at	0.5	5.4×10^{-27}
LEF1_221558_s_at	-0.09	6×10^{-2}
POU6F2_207450_s_at	0.68	1.9×10^{-58}

SP1_1553685_s_at	0.38	5.7×10^{-16}
SP1_214732_at	0.43	3.3×10^{-20}
SP1_224754_at	0.16	1.5×10^{-3}
SP1_224760_at	0.6	1.2×10^{-41}
TBP_203135_at	0.2	3.9×10^{-5}
TCF7_205254_x_at	0.51	6.1×10^{-29}
TCF7_205255_x_at	0.18	2.9×10^{-4}
TCF7L1_221016_s_at	0.5	1.6×10^{-27}
TCF7L2_212759_s_at	0.04	4.4×10^{-1}
TCF7L2_212761_at	-0.13	7.1×10^{-3}
TCF7L2_212762_s_at	-0.26	7.3×10^{-8}
TCF7L2_216035_x_at	-0.09	6.6×10^{-2}
TCF7L2_216037_x_at	0.06	2.2×10^{-1}
TCF7L2_216511_s_at	-0.11	2.7×10^{-2}
TCF7L2_236094_at	0.7	9.9×10^{-63}
TEAD2_226408_at	0.4	4.9×10^{-17}
TEAD2_238322_s_at	0.7	4.6×10^{-61}
TEAD2_238323_at	0.26	1.3×10^{-7}
TEAD2_243766_s_at	0.34	5.3×10^{-13}
TP53_201746_at	0.24	8.9×10^{-7}
TP53_211300_s_at	0.61	2.1×10^{-43}
